# Inhibition of Xanthine Oxidase by Allopurinol Prevents Skeletal Muscle Atrophy: Role of p38 MAPKinase and E3 Ubiquitin Ligases

**DOI:** 10.1371/journal.pone.0046668

**Published:** 2012-10-05

**Authors:** Frederic Derbre, Beatriz Ferrando, Mari Carmen Gomez-Cabrera, Fabian Sanchis-Gomar, Vladimir E. Martinez-Bello, Gloria Olaso-Gonzalez, Ana Diaz, Arlette Gratas-Delamarche, Miguel Cerda, Jose Viña

**Affiliations:** 1 Department of Physiology, University of Valencia, Fundacion Investigacion Hospital Clinico Universitario/INCLIVA, Valencia, Spain; 2 Laboratory “Movement Sport and Health Sciences”, University Rennes 2-ENS Cachan, Rennes, France; 3 UCIM, Faculty of Medicine, University of Valencia, Valencia, Spain; 4 Department of Pathology, University of Valencia, Valencia, Spain; McGill University, Canada

## Abstract

Alterations in muscle play an important role in common diseases and conditions. Reactive oxygen species (ROS) are generated during hindlimb unloading due, at least in part, to the activation of xanthine oxidase (XO). The major aim of this study was to determine the mechanism by which XO activation causes unloading-induced muscle atrophy in rats, and its possible prevention by allopurinol, a well-known inhibitor of this enzyme. For this purpose we studied one of the main redox sensitive signalling cascades involved in skeletal muscle atrophy i.e. p38 MAPKinase, and the expression of two well known muscle specific E3 ubiquitin ligases involved in proteolysis, the Muscle atrophy F-Box (MAFbx; also known as atrogin-1) and Muscle RING (Really Interesting New Gene) Finger-1 (MuRF-1). We found that hindlimb unloading induced a significant increase in XO activity and in the protein expression of the antioxidant enzymes CuZnSOD and Catalase in skeletal muscle. The most relevant new fact reported in this paper is that inhibition of XO with allopurinol, a drug widely used in clinical practice, prevents soleus muscle atrophy by ∼20% after hindlimb unloading. This was associated with the inhibition of the p38 MAPK-MAFbx pathway. Our data suggest that XO was involved in the loss of muscle mass via the activation of the p38MAPK-MAFbx pathway in unloaded muscle atrophy. Thus, allopurinol may have clinical benefits to combat skeletal muscle atrophy in bedridden, astronauts, sarcopenic, and cachexic patients.

## Introduction

Skeletal muscle atrophy is a debilitating consequence of multiple chronic diseases and conditions. It reduces treatment options and positive clinical outcomes as well as compromising quality of life and increasing morbidity and mortality [1]. Both systemic and local factors can initiate muscle atrophy. Systemic factors include increased myostatin and glucocorticoids; or a lack of anabolic hormones such as insulin or insulin-like growth factor-1 [2]. Local factors include muscle inactivity, muscle denervation or muscular dystrophies, and muscle ageing [3].

Muscle atrophy and weakness are linked to oxidative stress in several conditions: limb immobilization [4], hindlimb-unloading [5], [6], [7], [8], [9], chronic obstructive pulmonary disease [10] and sepsis [11]. Numerous cellular sites of ROS production exist in skeletal muscle, including NAD(P)H oxidase, nitric oxide synthase, heme oxygenase-1, mitochondria, and XO. The involvement of each of these oxidant sources in chronic ROS overproduction during muscular inactivity remains a topic of debate. Xanthine oxidoreductase (XOR) is an intracellular enzyme involved in purine catabolism. This enzyme catalyzes the reduction of hypoxanthine and xanthine to uric acid [12]. XOR exists in two interconvertible forms, xanthine dehydrogenase (XDH) and XO. In the oxidase form, molecular oxygen is used as the electron acceptor and hypoxanthine and xanthine are reduced to uric acid and superoxide. During activating conditions, XDH can be converted to XO via sulfhydryl oxidation or proteolytic cleavage. McCord et al. found that XO plays an essential role in ischemia-reperfusion injury [13]. Moreover, XO is a source of oxidant production in immobilized rats [14], in hindlimb unloading [8], in mechanical ventilation-induced diaphragmatic contractile dysfunction [15], and in cachexia [16]. However, the molecular mechanism(s) by which this enzyme elicits skeletal muscle atrophy remains unknown.

Allopurinol is a well-known inhibitor of XOR widely used in clinical practice [17]. We have previously reported that allopurinol prevents muscle oxidative damage during exhaustive physical exercise by inhibiting the MAPKinase/NF-κB cell signalling pathways [18], [19]. P38 MAPK mediates oxidative stress-sensitive cell signalling pathways and it has been suggested that chronic ROS overproduction plays a role in its activation during muscular inactivity [20], [21].

The loss in muscle mass during muscular inactivity is caused by both apoptosis of myonuclei [22] and by an imbalance between protein synthesis and degradation [23]. The discovery of two muscle-specific E3 ubiquitin ligases, Muscle atrophy F-Box (MAFbx; also known as atrogin-1) and Muscle RING (Really Interesting New Gene) Finger-1 (MuRF-1), prompted renewed expectation in identifying muscle-specific targets for therapeutic manipulation. MAFbx and MuRF-1 belong to the ubiquitin proteasome pathway, the primary pathway involved in intracellular protein degradation in skeletal muscle [24]. MAFbx and MuRF-1 regulate the degradation of key proteins involved in striated muscle growth and differentiation, including MyoD, calcineurin, troponin-I, titin and myosin heavy and light chains [25], [26].

The major aim of this study was to determine the mechanism(s) by which XO activation causes unloading-induced muscle atrophy and its possible prevention by allopurinol.

We have found that in unloaded muscles, XO activates the p38 MAPK which leads to the activation of the E3 ubiquitin ligases MAFbx. Both mechanisms trigger massive degradation of muscle proteins that is significantly prevented by allopurinol administration.

## Results

### Soleus muscle atrophy

**Figure 1:**
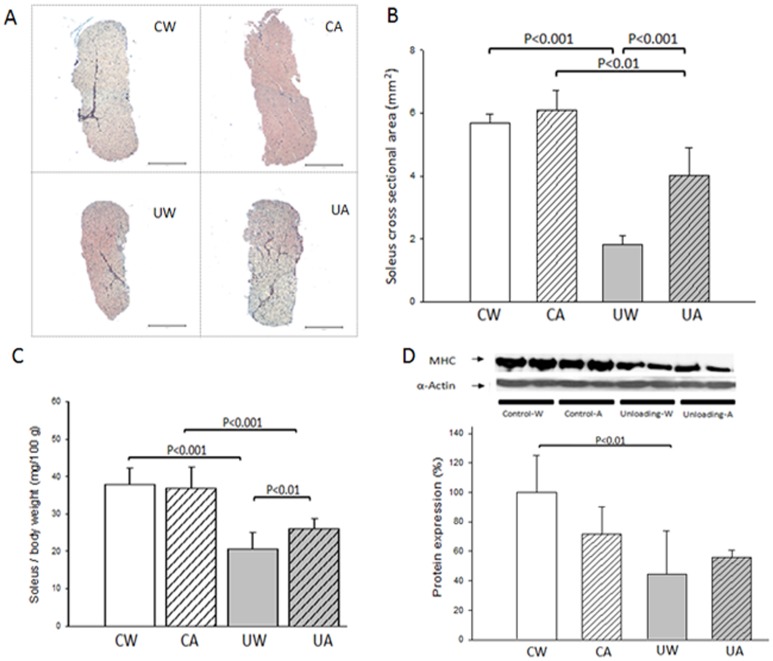
Allopurinol prevents soleus muscle atrophy after 14 days of hindlimb unloading in rats. (A) The reticulin staining (Original magnification, 4×) shows a significant decrease in the soleus muscle cross-sectional area after hindlimb unloading. Allopurinol partially prevents it (Scale bar, 1 mm). (B) Quantitative analyses were used to confirm histological findings, by comparing the cross-sectional area of the different groups: control rats with water (CW) (n = 3), control rats with allopurinol (CA) (n = 3), unloading rats with water (UW) (n = 3) and unloading rats with allopurinol (UA) (n = 3). (C) Soleus muscle to body mass ratio. Values are mean (±SD) in CW (n = 9), CA (n = 9), UW (n = 7), and UA (n = 7). (D) Western blot and densitometric analysis of slow skeletal muscle myosin heavy chain in CW (n = 3), CA (n = 3), UW (n = 3), and UA (n = 3). A two-factor ANOVA and post hoc Bonferroni's comparisons were used to identify significant differences.

We used different methods to determine the role of XO in the hindlimb unloading-induced soleus muscle atrophy. Hindlimb suspension for 14 days caused a significant decrease in the cross-sectional area of soleus muscle. Administration of allopurinol significantly prevented this decrement (Figure 1, panels A and B). Moreover, soleus muscle to body mass ratio was significantly reduced after 14 days of unloading (49%, p<0.001). This muscle atrophy was reduced to only 30% in the allopurinol treated animals (p<0.01 *vs* unloaded group with water) (Figure 1, panel C). We further tested the effect of inhibiting XO in the maintenance of muscle integrity by determining the protein content of an important component of the sarcomere, the slow Myosin Heavy Chain (MHC) protein. Figure 1, panel D, shows that after unloading there was a significant decrease in the protein content of MHC in soleus muscle. In this case we found only a partial prevention after treatment with allopurinol.

### XO activity

**Figure 2:**
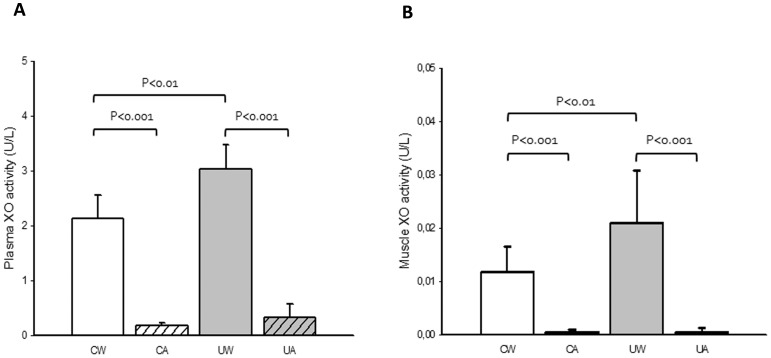
Hindlimb unloading activates plasma and soleus muscle XO. Prevention by allopurinol. Mean (±SD) results of XO activity in plasma (A) and soleus (B) of control with water (CW) (n = 9), control with allopurinol (CA) (n = 9), unloaded with water (UW) (n = 7) and unloaded with allopurinol (UA) (n = 7) rats. A two-factor ANOVA and post hoc Bonferroni's comparisons were used to identify significant differences.

Rat plasma (Figure 2A) and soleus muscle (Figure 2B) XO activity increased significantly after two weeks of hindlimb unloading. As expected, treatment with allopurinol completely inhibited XO activation both in plasma and in skeletal muscle.

### Oxidative stress

**Figure 3:**
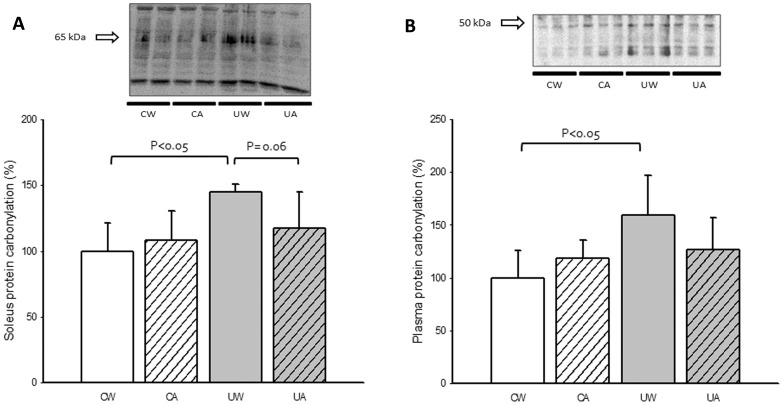
Effect of unloading and allopurinol treatment on soleus muscle and plasma protein carbonylation. Mean (±SD) results of soleus muscle carbonylated proteins (A) of control with water (CW) (n = 9), control with allopurinol (CA) (n = 9), unloaded with water (UW) (n = 7) and unloaded with allopurinol (UA) (n = 7) rats. Figure B represents carbonylation of low-molecular weight protein (less than 50 kDa) in plasma of the same animals. A two-factor ANOVA and post hoc Bonferroni's comparisons were used to identify significant differences.

As shown in Figure 3, hindlimb unloading caused a significant increase in skeletal muscle oxidative stress evidenced by carbonylation of soleus proteins. A protein(s) with a molecular weight of 65 kDa was significantly carbonylated in the muscle of unloaded rats (Figure 3A). We also determined plasma protein carbonylation in our animals. Figure 3B shows a significant increase in the carbonylation of low-molecular weight proteins (less than 50 kDa) in the samples of the unloaded animals that received water. A trend for allopurinol prevention of protein oxidation was found although it did not reach statistical significance either in the soleus or in plasma.

### Antioxidant enzymes

**Figure 4:**
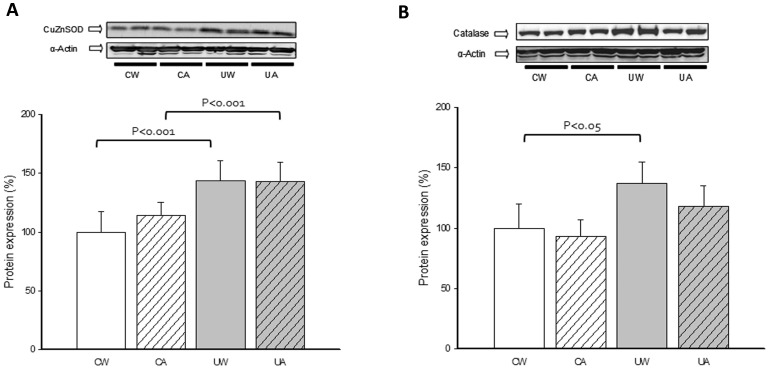
Effect of unloading and allopurinol treatment on the protein levels of soleus muscle antioxidant enzymes. Mean (±SD) results of CuZnSOD (A) and Catalase (B) protein levels in soleus muscle of control with water (CW) (n = 9), control with allopurinol (CA) (n = 9), unloaded with water (UW) (n = 7) and unloaded with allopurinol (UA) (n = 7) rats. A two-factor ANOVA and post hoc Bonferroni's comparisons were used to identify significant differences.

Hindlimb unloading increased the protein levels of the cytosolic CuZnSOD in the soleus muscle of the rats (Figure 4A). Catalase protein levels were also elevated in the soleus muscle of the unloaded animals (Figure 4B). Treatment with allopurinol did not prevent the unloading-induced increase of these antioxidant enzymes.

### p38 MAPK

**Figure 5:**
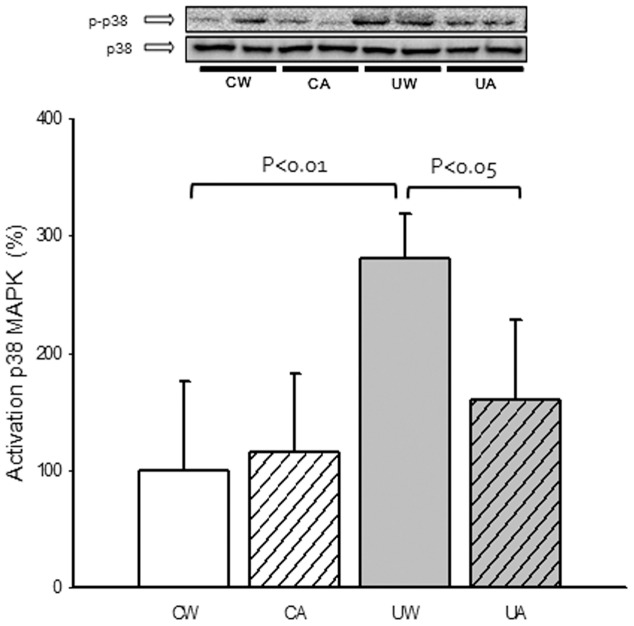
Effect of unloading and allopurinol treatment on the activation of p38 MAPK. Mean (±SD) results of cytosolic p38 MAPK in soleus muscle of control with water (CW) (n = 9), control with allopurinol (CA) (n = 9), unloaded with water (UW) (n = 7) and unloaded with allopurinol (UA) (n = 7) rats. Activation levels were expressed as the ratio between phosphorylated and total protein content. A two-factor ANOVA and post hoc Bonferroni's comparisons were used to identify significant differences.

p38 MAPK plays an important role in the coordination of the cellular responses to many stress stimuli, including oxidative stress [27]. Thus, we tested whether XO-derived ROS were involved in the activation of p38 MAPK in soleus muscle. Figure 5 shows a significant increase in the phosporylation of p38 MAPKinase in the soleus muscle of the unloaded animals. Allopurinol treatment completely prevented the p38 phosporylation. Total p38 protein levels were unaltered in the different experimental groups. The content of α-actin, a housekeeping protein marker in muscle, was not altered in the various treatment groups of rats (data not shown).

### E3 ubiquitin ligases MAFbx and MuRF-1

**Figure 6:**
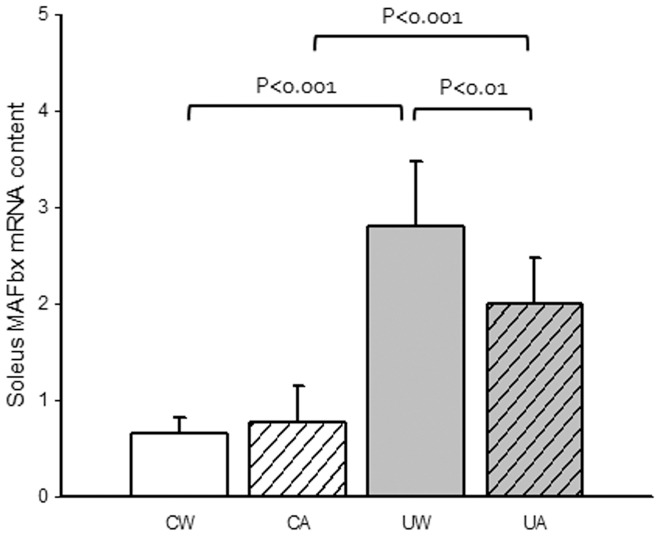
Effect of unloading and allopurinol treatment on the MAFbx levels in soleus muscle. Mean (±SD) results of MAFbx mRNA levels in soleus muscle of control with water (CW) (n = 9), control with allopurinol (CA) (n = 9), unloaded with water (UW) (n = 7) and unloaded with allopurinol (UA) (n = 7) rats. A two-factor ANOVA and post hoc Bonferroni's comparisons were used to identify significant differences.

To finally identify the mechanism by which allopurinol prevents the loss of muscle mass after hindlimb unloading, we determined the expression, in soleus muscle, of two well-known muscle specific E3 ubiquitin ligases namely, MAFbx and MuRF-1 [28]. As shown in Figure 6 we found a very significant increase in MAFbx mRNA expression in the soleus muscle of the unloaded animals and a significant prevention after treatment with allopurinol. It has been previously shown that p38 activity stimulates the expression of MAFbx [21]. Thus, our results are consistent with the idea that XO-derived radicals activate p38 and subsequently MAFbx ubiquitin ligase. This triggers protein degradation and muscle mass loss. All this is prevented by allopurinol. Regarding MuRF-1, our results show that hindlimb unloading induced its expression in the soleus muscle. However, although a tendency was found (p = 0.09), we find no prevention after allopurinol treatment (data not shown).

## Discussion

### XO is involved in the oxidative stress and soleus muscle atrophy associated to hindlimb unloading

Alterations in muscle play an important role in the most common diseases and conditions. For instance heart disease and cancer (two of the most prevalent chronic diseases) are often associated with rapid and extensive loss of muscle mass, strength, and metabolic function [29]. This loss of muscle mass is an important determinant of survival. Preservation of skeletal muscle is also critical during aging. Sarcopenia, the loss of muscle mass and strength that occurs with aging, is a widespread syndrome that has a devastating effect on quality of life and ultimately survival [30]. Here we demonstrate that treatment with allopurinol significantly prevents skeletal muscle atrophy after hindlimb unloading in rats. We found a remarkable decrease in the cross-sectional area of the rat's soleus muscle after unloading and a significant prevention after treatment with allopurinol (Figure 1, panel A and B). We also measured the soleus muscle atrophy by weighting the muscles, and we found a 20% prevention in the unloading-induced soleus atrophy under allopurinol treatment (p<0.01) (Figure 1, panel C). We further tested the effect of inhibiting XO in the maintenance of muscle integrity by determining the protein content of an important component of the sarcomere, the slow MHC protein. Figure 1, panel D, shows that after unloading there was a dramatic decrease in the protein content of MHC. In this case, we found only a partial prevention after treatment with allopurinol.

Eighteen years ago, Kondo and co-workers observed that muscle atrophy caused by hindlimb unloading was associated with an increase in lipid peroxidation and oxidized glutathione in rat skeletal muscle [4]. This first study was followed by substantial investigations in rodents confirming that muscular inactivity induces oxidative stress in skeletal muscle [31], [32]. Although the role of oxidative stress in muscle atrophy has been recently questioned in the hindlimb unloading animal model [33], several researchers have found that rodents supplemented with vitamin E [9], [34], Bowman-Birk inhibitor concentrate (soy protein with antioxidant properties) [6], and resveratrol [7], exhibit less oxidative damage and muscle atrophy in muscle disuse models (including hindlimg unloading). However, the source(s) of ROS production in the unloading models is still a subject of debate. In the pioneer work by Kondo and co-workers the authors pointed to XO as the main source of ROS production in the atrophied muscles [4]. Several years later Matuszczak et al. found that administration of allopurinol to mice had protective effects during hindlimb unloading [8]. Although, contrary to our results, the XO inhibitor did not decrease the atrophy caused by prolonged unloading, it blunted the contractile dysfunction in soleus muscles. We consider that it is possible that the higher dose of allopurinol used in our study could explain the differences between Matuszczak's data and ours. Although in both studies the drug dose was 50 mg^.^Kg^−1^, if we take into account the differences in the surface area between rats and mice, we administered 300 mg/m^2^ of allopurinol in our rat study while they administered 150 mg/m^2^ of allopurinol in their mice study. Body surface area has been recommended as the main basis for drug dosage, because the rate of metabolism or redistribution of a drug is proportional to the metabolic rate, which reflects heat losses that are generally proportional to the surface area [35]. More recently the group of Powers [15] showed that XO is involved in mechanical ventilation-induced oxidative injury and contractile dysfunction in the diaphragm. Respiratory muscle weakness produced by mechanical ventilation is due to diaphragmatic contractile dysfunction and atrophy and is directly linked to oxidative stress [36]. However, the molecular mechanisms involved in the XO-mediated skeletal muscle atrophy are not well understood. We have previously reported that inhibition of XO with allopurinol prevents the exercise-induced oxidative stress in skeletal muscle by inhibiting the MAPK/NF-κB signalling pathways [18], [37]. Thus, we aimed at determining the mechanism by which XO activation causes unloading-induced muscle atrophy and its possible prevention by allopurinol. We found a significant increase in plasma and soleus muscle XO activity associated to hindlimb unloading that was prevented completely by allopurinol treatment (See Figure 2). It is generally accepted that the Ca^+2^-activated proteases participate in the conversion of XDH into XO [13]. Kondo and co-workers reported an increase of intracellular Ca^+2^ in atrophic muscles using electron probe X-ray microanalysis [38]. Thus, the increased intracellular Ca^+2^ might enhance the enzyme conversion in the atrophied muscle through the activation of proteases [4].

We did not find a significant prevention of the protein oxidation induced by hindlimb unloading after allopurinol administration. Figure 3 shows a significant increase in the protein carbonylation in the plasma and soleus muscle in the unloaded animals. A trend for allopurinol prevention of protein oxidation was found although it did not reach statistical significance. The failure of allopurinol (or oxypurinol) to completely blunt the oxidative stress in skeletal muscle using different unloading models has been previously reported [8], [15].

A complex cytoprotective system that includes antioxidant enzymes is recruited against free radical damage. As previously observed by different research groups, unloading induced the expression of the antioxidant enzymes catalase and CuZnSOD in soleus muscle [8], [9]. These responses suggest that the muscle is adapting to oxidative stress. Allopurinol administration did not modulate the skeletal muscle antioxidant enzymes levels (See Figure 4). Thus, our data support the idea that XO is not the only source of ROS production in skeletal muscle during hindlimb unloading.

### Role of XO-derived ROS in the activation of the p38MAPK-E3 ubiquitin ligases signalling pathway during hindlimb unloading

p38 is a stress-activated protein kinase that responds to a variety of stimuli, including oxidative stress and TNF-α [39], and has been identified as a likely mediator of catabolic signaling in skeletal muscle [3], [21]. Thus, we determined the phosphorylation of p38 MAPK in the soleus muscle samples. We found a significant increase in p-p38 in the soleus of the unloaded animals that was prevented by allopurinol administration (See Figure 5). Our results are consistent with those reported by Childs et al. [40] which found a 128% increase in p38 phosphorylation after 10 days of hindlimb immobilization in rats. This was associated with a 38% decrease in soleus muscle mass over the same period. One year later Di Giovanni et al. [41] found that p38 phosphorylation was elevated in atrophic muscles of patients with acute quadriplegic myopathy and with neurogenic muscle atrophy. Other procatabolic conditions in which constitutive phosphorylation of skeletal muscle p38 has been shown to be elevated include Type 2 diabetes [42] and aging [43].

To identify the final mechanism by which allopurinol prevents the loss of muscle mass after hindlimb unloading, we determined the expression of two well known muscle specific E3 ubiquitin ligases involved in several *in vivo* models of skeletal muscle atrophy, MAFbx and MuRF-1 [28]. MAFbx and/or MuRF-1 mRNA are up-regulated following immobilisation in humans, hibernating squirrels, mice and rats [28]. The increases in MAFbx and MuRF-1 are associated with immobilisation-induced increases in proteasome dependent proteolysis. Treatments such as vitamin E [9] and resistance exercise [44] blunt the induction of the atrogenes following limb unloading.

Reid and co-workers showed in 2005 that p38 signalling promotes skeletal muscle atrophy through the expression of MAFbx in myotubes [21] but this was never tested in whole muscle *in vivo*. Figure 6 shows a very significant increase in MAFbx mRNA expression in the skeletal muscle of the unloaded animals and a partial prevention after treatment with allopurinol. Our data suggest that MAFbx gene is a downstream target of p38 MAPK signaling. This is consistent with the concept that pathologic elevation of p38 activity favours protein degradation and muscle atrophy. Our study does not identify the mechanism by which p38 increases MAFbx mRNA levels. It has been previously hypothesized that p38 MAPK can contribute to the activation of Foxo (forkhead-type) transcription factors that have been identified as an essential regulator of MAFbx in skeletal muscle [21], [45].

Novel potential atrogenes have been identified using microarray analysis [46]. In 2009 it was shown that induction of Cbl-b (a RING-type ubiquitin ligase) *in vivo* was required for the loss of muscle mass in response to unloading [47]. Thus, apart from MuRF-1 and MAFbx, this ubiquitin ligase should be also properly studied in future experimental designs.

**Figure 7:**
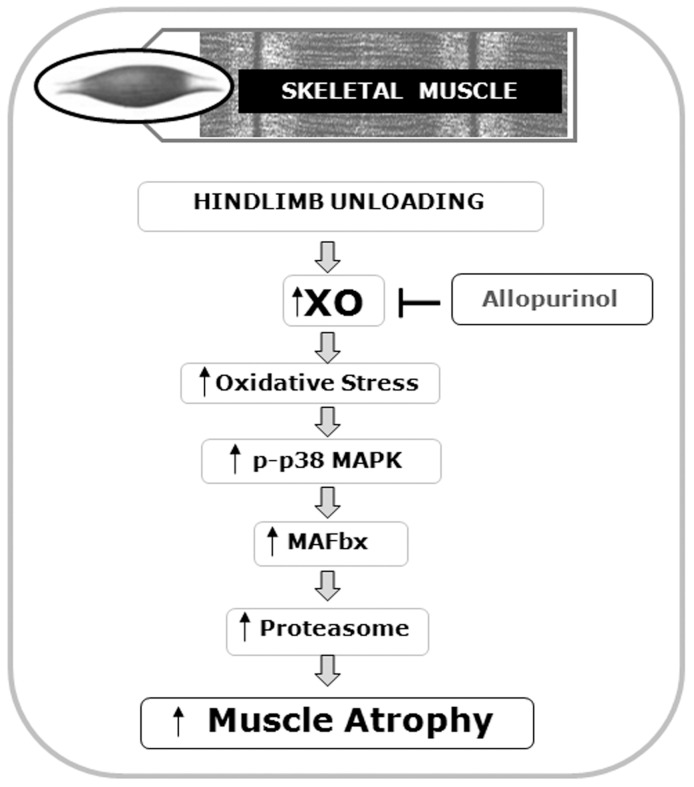
Allopurinol prevents the loss of muscle mass during hindlimb unloading via inhibition of p38 MAPK and the E3 ubiquitin ligase Atrogin1/MAFbx.

Collectively, our data suggest that XO is involved in the activation of the p38MAPK-MAFbx pathway in unloaded muscle atrophy. We have shown that allopurinol treatment, during unloading, completely prevents the activation of XO and partially prevents the loss of muscle mass in soleus muscle. One of the main oxidative-stress signalling cascades, p38-MAPKinase, is activated after 14 days of hindlimb unloading. This activation coincides with the increase in the mRNA expression of the proteolytic E3 ubiquitin ligases MuRF-1 and MAFbx in soleus muscle. Allopurinol treatment significantly prevents the activation of the p38 MAPK-MAFbx pathway but not MuRF-1 one. A schematic interpretation of our results is in Figure 7.

Recent findings show that inhibition of XO reduces oxidative stress and improves skeletal muscle function in response to electrically-stimulation in aged mice [48]. Hyperuricaemia has been observed to be a negative prognostic marker in end-stage cancer patients [49]. Moreover, elevated uric acid levels, resulting from up-regulated XO activity, have been shown to have predictive value for mortality in chronic heart failure [50]. Our data support the idea that XO is not the only source of ROS production in the skeletal muscle during hindlimb unloading, but point out the potential benefit of allopurinol administration for bedridden, astronauts, sarcopenic and cachexic patients.

## Materials and Methods

### Animal care and protocol

The experimental protocol was approved by the Committee on Ethics in Research of the Faculty of Medicine, University of Valencia.

Thirty-two young male Wistar rats (∼300 g) were housed in a temperature-controlled room (24±2°C) with a light-dark cycle (12∶12 h). After one week of acclimation, the rats were assigned randomly to one of four experimental conditions: freely ambulating with water (CW) (n = 9); freely ambulating treated with allopurinol (CA) (n = 9); hindlimb unloaded with water (UW) (n = 7); or hindlimb unloaded treated with allopurinol (UA) (n = 7).

Allopurinol (Sigma Chemical, St. Louis, MO) was administered to the animals via the drinking water. An initial concentration of 25.6 mg^.^dl^−1^ was adjusted daily to achieve a target of 50 mg^.^kg^−1.^day^−1^ as calculated for each animal based on daily water intake. After a 48 h allopurinol pre-treatment, hindlimb unloading was accomplished using the method of Morey-Holton and Globus, a standard model used to unload antigravity muscles [51]. In brief, Elastoplast® tape was wrapped along the long axis of each animal's tail. A metal clip on the tape was attached to a nylon monofilament line via a stainless steel swivel. The distal end of the nylon line was attached to an overhead support and was shortened to suspend the animal in a 45° head-down tilt position. The swivel enabled the animal to explore the cage (360° range of motion) and obtain food and water freely. Animals were observed daily for changes in appearance and activity. Each animal was weighed; food, water, and allopurinol intakes were recorded; and the angle of hindlimb suspension was adjusted if necessary. After 14 days of conditioning, each animal was deeply anesthetized while hindlimb-suspended. The animal then was removed from the suspension device. Blood was obtained by venous puncture into heparin-containing tubes. Soleus muscles were excised, weighed, frozen in liquid nitrogen, and stored at −80°C until analysis. Rats were then euthanized by an overdose of the anaesthetic.

### Xanthine oxidase activity

XO activity was measured in plasma and soleus muscle by the fluorimetric method described in Beckman et al. [52]. Briefly, isoxantopterine formation from pterine was followed fluorometrically as previously described (excitation at 345 nm and emission at 390 nm) [53].

### Immunoblotting

Aliquots of muscle lysates (40–120 µg of proteins) were resolved on 12.5% and 15% SDS-PAGE gels depending on the molecular weight of the protein of interest [54]. Proteins were then transferred to nitrocellulose membranes, which were incubated overnight at 4°C with appropriate primary antibodies: anti-CuZnSOD (1∶5000, NovusBio), anti-Catalase (1∶5000, Sigma Aldrich, Missouri), anti-α-actin (1∶700, Sigma Aldrich), anti-p38 MAPK, anti-Phospho-p38 MAPK (1∶1000, Cell Signaling), and anti-MHC (1∶10000, Chemicon Millipore). Thereafter, membranes were incubated with a secondary antibody for 1 h at room temperature. Specific proteins were visualized by using the enhanced chemiluminescence procedure as specified by the manufacturer (Amersham Biosciences, Piscataway, NJ). Autoradiographic signals were assessed by using a scanning densitometer (BioRad, Hercules, CA).

### Histology

The soleus muscle samples were formalin-fixed and paraffin-embedded in blocks. Nine serial transverse sections of 5 µm were obtained from each sample using a LEICA RM 2245 microtome and were mounted on glass slides, three cuts in each. The cuts were performed in the wider part of the soleus muscle. Subsequently, the sections were stained using the Gomori method for the reticulin stain. All the histological sections were visualized using a LEICA DMD 108 light microscopy. These samples were used to measure the cross-sectional areas of each muscle, determined per field using the Img.Pro.Plus.6 software from captured images at 4×.

### RNA isolation, reverse transcription and PCR

Total RNA was isolated with the RNeasy® Fibrous Tissue Mini Kit (Quiagen, Madrid, Spain) following the manufacturer's instructions and the final pellets were resuspended in 20 µL of nuclease-free H_2_O. The purity of the samples was assessed determining the 260/280 nm ratio, which was always above 1.9. We synthesized cDNA from 1 µg of RNA using random hexamer primers and the High Capacity cDNA Reverse Transcription Kit (Applied Biosystems, Madrid, Spain). Reverse transcription conditions comprised an initial incubation step at 25°C for 10 minutes to allow random hexamers annealing, followed by cDNA synthesis at 37°C for 120 minutes, and final inactivation step for 5 minutes at 95°C. Real-time PCR was performed with an ABI 7900 sequence-detection system (Applied Biosystems, Madrid, Spain). Primers for amplifying specific fragments of the genes were obtained from Thermo Fisher Scientific GmbH (Ulm, Germany). The specific primers used in our experiments are shown in Table 1.

**Table 1:** Primer sequences used for real-time PCR.

	Sense	Anti-sense
MAFbx	5′- GCCTGAACTACGATGTTGCAG - 3′	3′- GCTGGTCTTCAAGAACTTTC - 5′
MuRF1	5′- AGGTGCCTACTTGCTCCTTGT - 3′	3′ - GCTGTTTCCACAAGCTTGGTC - 5′
Cyclophilin	5′- GTGGCAAGTCCATCTACGGAG - 3′	3′- CCACAGTCGGAGATGGTGATC-5′

Real-time PCR was performed in duplicate in a total reaction volume of 20 µL using Maxima™ SYBR green/ROX qPCR Master Mix (Fermentas, Madrid, Spain). The thermal cycling protocol was as follows: initial denaturation for 10 minutes at 95°C was followed by 40 cycles of 10 seconds at 95°C, 10 s at 62°C and 10 s at 72°C. The fluorescence signal was measured at the end of each extension step at 72°C. At the end of each reaction, a melting curve analysis was performed to confirm that only the specific products were amplified. The threshold cycle (Ct) was converted to a relative gene expression by using a standard curve. For each sample, the expression of the target gene was normalized with the cyclophilin mRNA content.

### Protein carbonylation

Oxidative modification of total proteins in plasma and skeletal muscle fractions were assessed by immunoblot detection of protein carbonyl groups using the ‘OxyBlot’ protein oxidation kit (Intergen) as previously described [55]. Approximately 20 µg of total protein was loaded onto gels and electrophoretically separated. Antibody anti-dinitrophenylhydrazone was purchased from Intergen Company (Purchase, NY). The procedure to quantify total protein carbonyls with the OxyBlot kit was densitometry of the blotting and of the Ponceau red staining (data not shown), followed by finding the ratio between the total density in the oxyblot and the total density in the Ponceau red staining. Specific proteins were visualised by using the enhanced chemiluminescence (ECL) procedure as specified by the manufacturer (Amersham). Autoradiographic signals were assessed using a BioRad scanning densitometer.

### Statistics

Results are expressed as means ± SDs. Normality of distribution was checked with the Kolmogorov test, and homogeneity of variance was tested by Levene's statistics. To test for statistically significant differences between the groups, two-way ANOVA was used. When significant F-ratios were observed, a Bonferroni multiple comparison's test was applied to test individual means. Statistical significance was assumed at p<0.05.
